# Supplementary material for: Content-rich biological network constructed by mining PubMed abstracts
Source: BMC Bioinformatics. 2004 Oct 8;5:147. doi: 10.1186/1471-2105-5-147 (PMC528731; doi:10.1186/1471-2105-5-147)
Supplement: Additional File 5 — The original Chilibot query results of the term "long-term potentiation (LTP)" and 22 other terms, limiting the latest references analyzed to the years 1990, 1995, 2000, and 2004. [file 1471-2105-5-147-S5.bz2 › chilibotAdditionalFile5/ltp1995/html/AMPA_TAU.html]

 


 **AMPA** and **TAU** 
  
Found 8 abstracts in PubMed,  **8 abstracts were retrieved and analyzed**.  


---

 Search Google  |
 PDF files only 
|  EDU domain only 

---

**Interactive relationship** (e.g. stimulation, inhibition, etc)

- In favor of the existence of glutamate receptors of the  **AMPA**  type on dopaminergic nerve terminals, the stimulatory effect of  **AMPA**  5 x 10 5 M on 3H dopamine release was antagonized by dinitroquinoxaline dione, 6 cyano 7 nitro quinoxaline dione,  **tau**  D glutamyl amino methyl sulphonate and  **tau**  D glutamyl glycine tested at 10 4 M.  Ref: 1682483 J Pharmacol Exp Ther, 1991

- :-)
